# Attenuation of N-nitrosodimethylamine induced hepatotoxicity by *Operculina turpethum* in Swiss Albino mice

**Published:** 2014-01

**Authors:** Veena Sharma, Manu Singh

**Affiliations:** 1 Department of Bioscience and Biotechnology, Banasthali University, Banasthali, Rajasthan, India; 2 Department of Bioscience and Biotechnology, Banasthali University, Banasthali, Rajasthan, India

**Keywords:** Hepatotoxicity, Liver, N-nitrosodimethylamine *Operculina turpethum*

## Abstract

***Objective(s):*** To appraise the antihepatotoxic efficacy of ethanolic extract of *Operculum turpethum* root on the liver of Swiss albino mice.

***Materials and Methods:*** Hepatic fibrosis was induced in adult male albino mice through intraperitoneal administrations of N-nitrosodimethylamine (NDMA) at the concentration of 10 mg/kg body weight. The liver toxicity and therapeutic effect of the plant ethanolic extract was assessed by the analysis of liver marker enzymes and antioxidant enzymes and liver histopathological studies.

***Results:*** Hepatotoxicity was manifested by significantly decreased (*P*<0.01) levels of the activities of the enzymatic and non enzymatic antioxidants such as superoxide dismutase, catalase, GSH and increased levels of cholesterol, AST, ALT, ALP and lipid peroxidation. The plant extract significantly restored the antioxidant enzyme level in the liver and exhibited significant dose dependent curative effect against NDMA induced toxicity which was also supported by histopathological studies of the liver.

***Conclusion:*** *O. turpethum* manifested therapeutic effects by significantly restoring the enzymatic levels and reducing the hepatic damage in mice. This work intends to aid researchers in the study of natural products which could be useful in the treatment of liver diseases including cancer.

## Introduction

Liver is one of the largest organs in human body and the chief site for metabolism and detoxification of the exogenous and endogenous challenges, like xenobiotics, drugs, viral infections and chronic alcoholism (1). Xenobiotic-induced liver diseases are major health problems that challenge not only health care professionals but also the pharmaceutical industry and drug regulatory agencies (2). The manifestations of xenobiotic- induced hepatotoxicity are highly variable, ranging from asymptomatic elevation of liver enzymes to culminate hepatic failure (3).

Free radicals or oxidative injury are critically involved in various pathological conditions such as cancer, neurological disorder, arthritis, inflammation and liver diseases. There are still no specific treatments in modern medicine that protect the liver against damage or help to regenerate hepatic cells (4). 

There is a growing interest in herbal remedies because of their effectiveness, minimal side effects in clinical experience and relatively low cost. Herbal drugs or their extracts are prescribed widely, even when their biological active compounds are unknown (5). Therefore, studies on plant extracts are useful to explore their efficacy, mechanisms of action and safety. Because of this fact, efforts have been made to find suitable curative agents for the treatment of liver diseases among natural products (6). Many active plant extracts are frequently used to treat a wide variety of clinical diseases including liver diseases. Therefore, search for effective and safe drugs for liver disorders continues to be an important area of scientific research.

**Figure 1:**
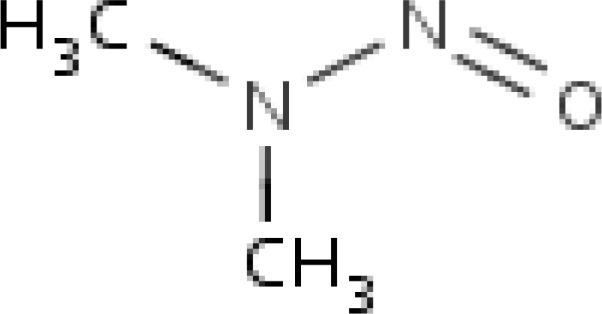
Structure of N-Nitrosodimethylamine

N-Nitrosodimethylamine (NDMA) is known to cause perturbations in the nuclear enzymes involved in deoxyribonucleic acid (DNA) repair/replication. Chemical-induced liver injury depends mostly on the oxidative stress in hepatic tissue and underlies the pathology of numerous diseases, including cancer. Experimental, clinical and epidemiological studies have provided evidence supporting the role of reactive oxygen species in the etiology of toxicity. NDMA is a member of a family of extremely potent carcinogens, the *N*-nitrosamines (7) (Figure 1). 

NDMA can occur in drinking-water through the degradation of dimethylhydrazine, a component of rocket fuel, as well as from several other industrial processes. It is also a contaminant of certain pesticides and found as disinfection by-product in waste water treatment plants. So, there is a great chance for NDMA to reach human biological systems (8). Hepatic fibrosis is characterised by excessive accumulation of connective tissue components, especially matured collagen fibres in the extracellular matrix of the liver. Data on therapeutic potential of synthetic drugs to treat liver cirrhosis are scarce and the toxic side effects may remain a persistent risk. Therefore, drugs from natural sources are being adopted to treat liver diseases (9).

The plant *Operculina turpethum*, which is commonly known as trivit, has been used as a folk medicine in many countries to treat jaundice, rheumatism, chronic gout, piles, tumours, obesity and many other diseases including liver disorders (10). Traditionally, roots or stem bark of this plant are used for medicinal purposes. The plant contains a glycosidic resin, which has the insoluble glycoside turpethin and two ether soluble glycosides. In spite of its various medicinal uses, no systematic studies regarding its pharmacological effect as a chemotherapeutic agent have been reported in the literature (11). Therefore, the aim of this investigation was to evaluate the antihepatotoxic activity of the ethanolic extract of *O. turpethum *root against NDMA induced liver toxicity in mice.

## Materials and Methods

***Chemicals***

TBA, TCA, HCl, pyrogallol, H_2_O_2_, triton-x, BSA, copper sulphate, ascorbic acid, thiourea, etc. All chemicals used in this study were of analytical reagent grade and were purchased from reliable producers (SRL (India), MERCK, RANBAXY, HIMEDIA). NDMA was purchased from SIGMA. 

***Animal care and monitoring***

Healthy male Swiss albino mice (*Mus musculus*) (4-6 weeks old, weighing 20-30 g) were procured from CCS Haryana Agricultural University (Hisar, India). They were housed under standard laboratory conditions of light (12:12 hr L: D cycle), temperature (23 ± 2°C) and relative humidity (55 ± 5%). Animals had free access to standard food pellet diet (Hindustan Lever Limited: metal contents in parts per million dry weight: Cu 10.0, Zn 45.0, Mn 55.0, Co 5.0, Fe 75.0) and drinking water *ad libitum *throughout the study. 

***Plant material***

*O. turpethum* was collected from the pharmacological garden of CCSHAU Hisar, Haryana, India in November 2011.The plant was identified with the help of available literature and authenticated by the botanist of Krishi Vigyan Kendra Rohtak, Haryana, India.

***Preparation of ethanolic extract***

Freshly collected *O.turpethum *roots were dried in shade and coarse powder was extracted. Dried powdered material was placed in the soxhlet thimble with 80% ethanol in 500 ml flat bottom flask. It was further refluxed for 18 hr at 80°C for two days. Collected solvent was cooled and poured in a glass plate. The filtrate was dried in hot air oven below 50°C for 48 hr and kept in dissector for 2 days. The yield of the extract was 12.5% w/w of powdered plant material for further exploration. Collected dried extract was stored at 5^0^C in air tight containers.

***Ethic clearance***

The animal experiments were carried out according to the guidelines of Committee for the Purpose of Control and Supervision of Experiments on Animals (CPCSEA). The Institutional Animal Ethics Committee approved experimental design performed in this study for the use of Swiss albino mice as the animal model for the study.

***Treatment regime***

The male Swiss albino mice *(Mus musculus) *were randomly selected from laboratory stocks and were placed into various groups.

Group 1 - Control

Group 2 - NDMA treated (10 mg/kg body weight)

Group 3 - NDMA + OTE (300 mg/kg body weight)

Group 4 - NDMA+ OTE (400 mg/kg body weight)

Group 5- OTE (300 mg/kg body weight)

Group 6- OTE (400 mg/kg body weight)

Group 7- NDMA + Standard antioxidant (BHA1%)

Group 8- BHA (1%)

The doses of the plant extract, NDMA and standard antioxidant were decided on the basis of previously published reports (12). NDMA was given on three consecutive days of each week for three successive weeks along with the plant extract simultaneously. 

***Biochemical assays***

After 21 days, the mice were fasted overnight and then sacrificed under light ether anesthesia. Liver lobules were dissected out, washed immediately with ice-cold saline to remove blood, and the wet weight was noted and then stored at -80°C for various biochemical assays and histological studies.

***Preparation of liver homogenate***

The liver was minced and homogenized (10% w/v) in ice-cold 0.1 M sodium phosphate buffer (pH 7.4). The homogenate was centrifuged at 10,000 rpm for 15-20 min at 48°C twice to get the enzyme fraction. The supernatant was used for biochemical assays (13). 

***LPO (Lipid peroxidation)***

LPO was estimated colorimetrically by measuring malondialdehyde (MDA) formation as described by Nwanjo and Ojiako (14). In brief, 0.1 ml of homogenate was treated with 2 ml of a 1:1:1 ratio of TBA-TCA-HCl (TBA 0.37%, TCA 15%, HCl 0.25 N) and placed in water bath at 65°C for 15 min, cooled, and centrifuged at 5,000 rpm for 10 min at room temperature. The optical density of the clear supernatant was measured at 535 nm against the reference blank. The MDA formation was calculated using the molar extinction coefficient of thiobarbituric acid reactants (TBARS; 1.56 x105 l/mole cm^-^1). The production of LPO was expressed as nmol of MDA formed per g of tissue.

***Superoxide dismutase (SOD)***

Hepatic SOD activity was assayed according to the method of Marklund and Marklund (15). For the control, 0.1 ml of 20 mM pyrogallol solution was added to 2.9 ml of Tris buffer and mixed, and reading was taken at 420 nm after 1.5 and 3.5 min. The absorbance difference for 2 min was recorded and the concentration of pyrogallol was adjusted in such a way that the rate of change in absorbance per 2 min was approximately 0.020-0.023 optical density units. Liver extract (200 ml) was treated with 10 ml of 25% triton X-100 and kept at 48°C for 30 min. To 2.8 ml of Tris buffer, 0.1 ml of treated sample was added and mixed. The reaction was started by adding 0.1 ml of adjusted pyrogallol solution (as for control). Reading was done at 420 nm after 1.5 and 3.5 min and the difference in absorbance was recorded. The enzyme activity was expressed as U/ml of liver extract and 1 U of enzyme is defined as the enzyme activity that inhibits auto-oxidation of pyrogallol by 50%.

***Catalase (CAT)***

Catalase (CAT) activity was estimated based on the method of Aebi (16). Liver extract (100 ml) was treated with ethanol (10 ml) and placed on an ice bath for 30 min. To this, 10 ml of 25% triton X-100 was added and again kept for 30 min on ice. To 200 ml phosphate buffer (0.1 M), 50 ml of treated liver extract and 250 ml of 0.066 M H_2_O_2_ (prepared in 0.1 M phosphate buffer, pH 7.0) were added in a cuvette. The decrease in optical density was measured at 240 nm for 60 sec. The molar extinction coefficient of 43.6 cm-1 was used to determine CAT activity. One unit of activity is equal to the moles of H_2_O_2_ degraded/min/mg protein.

***Aspartate*** ***aminotransferase*** ***(AST)*** ***and*** ***alanine***

***aminotransferase (ALT)***

Activities of aspartate aminotransferase (AST) and alanine aminotransferase (ALT) were assayed by the method of Reitman and Frankel (17). In brief, 0.2 ml of sample and 0.5 ml of substrate solution (for AST: aspartate and 2-ketoglutarate; for ALT: alanine and 2-ketoglutarate) were incubated at 37^o^C for 60 min for AST and 30 min for ALT. After incubation, 0.5 ml of DNPH solution was added to arrest the reaction, which was kept for 20 min at room temperature. To this, 1 ml of 0.4 N NaOH was added and absorbance was read at 510 nm. Activities were expressed as U/l.

***Reduced glutathione (GSH)***

Hepatic reduced glutathione (GSH) level was determined by the method of Ellman modified by Jollow *et al *(18). Sulphosalicylic acid (0.5 ml 10%) was added to mixture of 0.4 ml homogenate and 0.6 ml of distilled water as protein precipitant. Supernatant (0.5 ml) was mixed with the reaction mixture of 4.5 ml of 0.5 M Tris-buffer and 0.5 ml of 10mM DTNB and the absorbance was measured immediately at 412 nm. The GSH contents were calculated using GSH as standard and expressed as mM/g tissue.

***Protein***

Protein content was determined by the method of Lowry *et al*, (19) and bovine serum albumin as the standard.

***Cholesterol***

Cholesterol level was determined by the method of Zak (20) with cholesterol as the standard.

***Alkaline phosphatise (ALP)***

Activities of alkaline phosphatase (ALP) were determined according to the protocol described in a laboratory practical manual (21). Substrate solution (3 ml) was incubated at 37°C for 15 min and then 0.5 ml of sample was added. It was mixed well and immediately 0.05 ml of the mixture was removed and mixed with 9.5 ml of 0.085 N NaOH. This corresponded to zero time assay (blank). The remaining solution (substrate-enzyme) was incubated for 15 min at 37°C and then 0.5 ml was drawn and mixed with 9.5 ml of 0.085 N NaOH. Absorbance was measured at 405 nm against the reference blank. Specific activities were expressed as µmoles of p-nitrophenol formed per min per g tissue.

**Table 1:** Effect of ethanolic extract of* Operculum turpethum *(OTE) root on the levels of liver marker enzymes AST, ALT and ALP against NDMA induced hepatic damage

Groups	AST (U/l)	ALT (U/l)	ALP (µml/min/g)
Control	46.620±0.231^#^	38.486±0.024^#^	67.287±0.039^#^
NDMA (10 mg/kg)	69.164±0.011*	54.039±0.177*	86.164±0.011*
NDMA+OTE (300 mg/kg)	58.189±0.005^#^	48.083±0.012^#^	75.189±0.005^#^
NDMA+OTE (400 mg/kg)	53.880±0.193^#^	43.048±0.024^#^	69.880±0.191^#^
OTE (300 mg/kg)	46.553±0.193*	38.102±0.005^*^	68.819±0.003*
OTE (400 mg/kg)	47.242±0.009*	38.037±0.012*	68.242±0.010*
NDMA+BHA (1%)	49.270±0.014^#^	39.126±0.014^#^	68.270±0.014^#^
BHA (1%)	45.135±0.051*	37.140±0.005^*^	66.001±0.082*

Values are expressed as mean ± S.E.M for six mice in each group. * P < 0.01 *vs*. control and ^#^* P <*0.01 *vs*. NDMA treated group

AST (Aspartate aminotransferase), ALT (Alanine aminotransferase), ALP (Alkaline phosphatase).

***Histopathological studies***

The liver lobules were excised and fixed in 10% formalin, stained with haemotoxylin and eosin and then observed under microscope for degeneration, fatty changes, necrotic changes and evidence of hepatotoxicity (22). Results of the histopathological studies are shown in the (Figures 4-7).

***Statistical analysis***

Data are expressed as the mean±SEM. The data was analyzed by analysis of variance (ANOVA) using the Statistical Package for the Social Sciences (SPSS 11). 

## Results

***Effect of O. turpethum root extract on lipid peroxidation***

**Figure 2:**
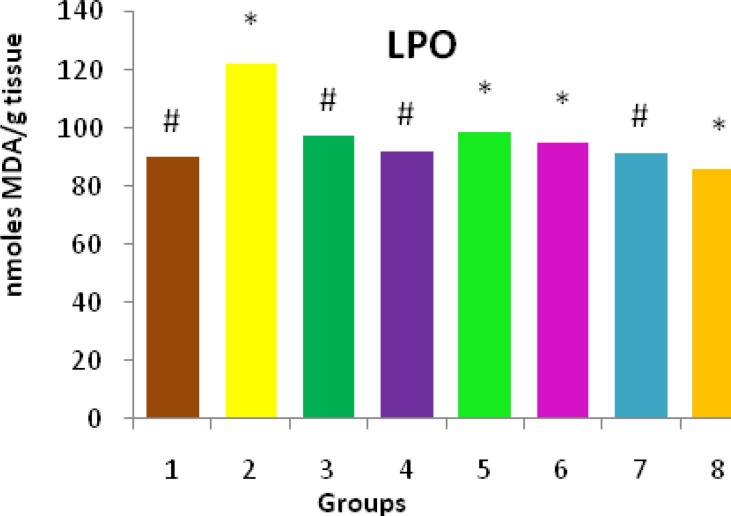
Effect of ethanolic extract of* Operculum turpethum* on the lipid peroxidation in different groups of mice showing significant changes in plant treated groups with *P-*value of *(*P<*0.01) as compared to the control and #(*P*<0.01) as compared to NDMA treated groups

The level of TBARS as an index of lipid peroxidation, a degradative process of membrane lipids, in liver tissue of NDMA treated mice was significantly (*P<*0.01) elevated (122.15±0.32) when compared to control animals (89.90±0.50). The remarkable increase in lipid peroxides in liver tissue during NDMA administration indicates the formation of reactive oxygen species (ROS), which play a major role in cell injury and pathogenesis of hepatic fibrosis. Lipid peroxidation level was restored towards its normal value by treatment with the *O.turpethum *extract on NDMA induced toxicity (Figure 2). 

***Effect of O. turpethum root extract on liver marker enzymes***

 Biochemical parameters in the control and various experimental groups are mentioned in Table 1. Administration of intraperitoneal NDMA to mice caused liver damage as indicated by a significant increase in liver enzymes AST, ALT, activity compared to control mice. Elevated levels of these enzymes are indicative of cellular leakage and loss of functional integrity of cell membrane in liver. Treatment of animals with the plant extract significantly (*P*<0.01) recovered the normal range of enzymes.

***Effect of O. turpethum root extract on antioxidant enzymes***

*On liver SOD level*

Effect of NDMA alone and co-treatment with *Oturpethum *extract on SOD activity has been shown in Table 2. SOD activity in NDMA treated liver tissue (2.501±0.027) was reduced markedly compared to the control group (9.112±0.004) and co-treatment with *O.turpethum *at the dose of 400 mg/kg body weight in the NDMA treated mice significantly (*P<*0.01) recovered that SOD depletion.

*On liver CAT level*

CAT activity in the NDMA treated group showed marked reduction compared to normal group (3.706±0.177 in NDMA treated group *vs*. 8.153±0.009 in control group). Catalase (CAT) is an enzymatic antioxidant widely distributed in all animal tissues and its highest activity is found in the liver. CAT decomposes hydrogen peroxide and protects the tissue from highly reactive hydroxyl radicals. Therefore, the reduction in the activity of these enzymes may result in a number of deleterious effects. As shown in Table 2, co-treatment with *O. turpethum *at the dose of 400 mg/kg body weight in the NDMA treated mice significantly (*P<*0.01) restored the CAT activity.

**Table 2:** Effect of ethanolic extract of *Operculum turpethum *root on the levels of antioxidant enzymes SOD, CAT, total protein, total cholesterol against NDMA induced hepatic damage

Groups	SOD (U/ml)	CAT (nanomoles/min/mg)	Cholesterol (mg/g)	Protein (mg/g)
Control	9.112±0.004^#^	8.153±0.009^#^	28.646±0.005^#^	86.287±0.039^#^
NDMA (10 mg/kg)	2.501±0.027*	3.706±0.177*	40.061±0.012*	60.830±0.190*
NDMA+OTE (300 mg/kg)	4.128±0.002^#^	5.150±0.012^#^	33.804±0.194^#^	72.522±0.189^#^
NDMA+OTE (400 mg/kg)	6.121±0.006^#^	6.048±0.024^#^	28.760±0.010^#^	79.213±0.001^#^
OTE (300 mg/kg)	8.106±0.011*	8.012±0.002*	27.331±0.010*	86.019±0.003*
OTE (400 mg/kg)	9.022±0.001*	8.037±0.012*	27.019±0.002*	86.142±0.009*
NDMA+BHA (1%)	6.631±0.001^#^	7.126±0.014^#^	32.129±0.002^*^	85.703±0.198^#^
BHA (1%)	9.108±0.001^*^	9.140±0.005*	28.114±0.006*	88.985±0.031*

Values are expressed as mean ± S.E.M for six mice in each group. *P < 0.01 *vs*. control and #*P<*0.01 *vs*. NDMA treated group

SOD:Superoxide dismutase, CAT: Catalase

*On total protein and total cholesterol level*

NDMA enhanced the levels of cholesterol in mice (40.061±0.012) and depleted the protein level (60.830±0.190) which were significantly recovered as (28.760±0.010) and (79.213±0.001**)** with the simultaneous dosing of the extract at 400 mg/kg body weight in the NDMA treated mice as shown in Table 2.

***Effect of O. turpethum on GSH level***

**Figure 3:**
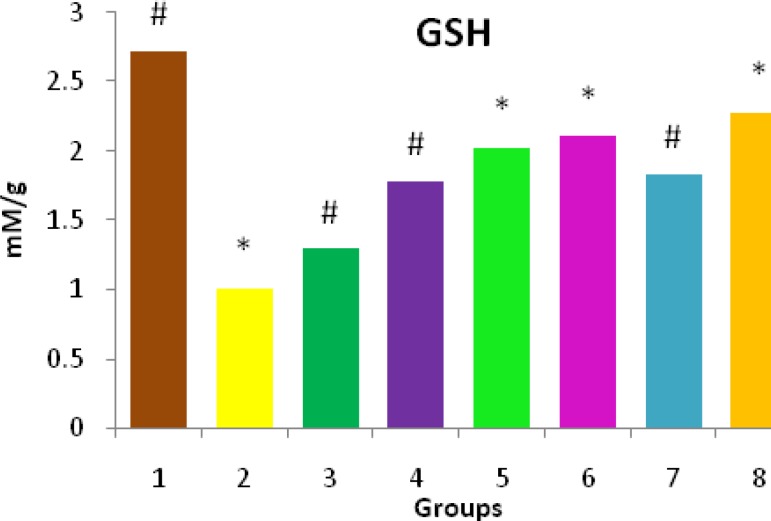
Effect of ethanolic extract of *Operculum turpethum* root on the GSH levels in different groups of mice showing significant changes in different plant treated groups with *P-*value of *(*P*<0.01) as compared to the control and # (*P*<0.01) as compared to NDMA treated group

GSH level as measured in the liver tissue of all the experimental groups has been shown in Figure 3. NDMA administration caused massive reduction in liver GSH level (1.004±0.001 *vs*. 2.720±0.002 in normal mice). Administration of the ethanolic extract at the dose of 400 mg/kg body weight simultaneously with NDMA significantly (*P*<0.01) elevated that reduction in animals.

***Histopathological studies***

**Figure 4:**
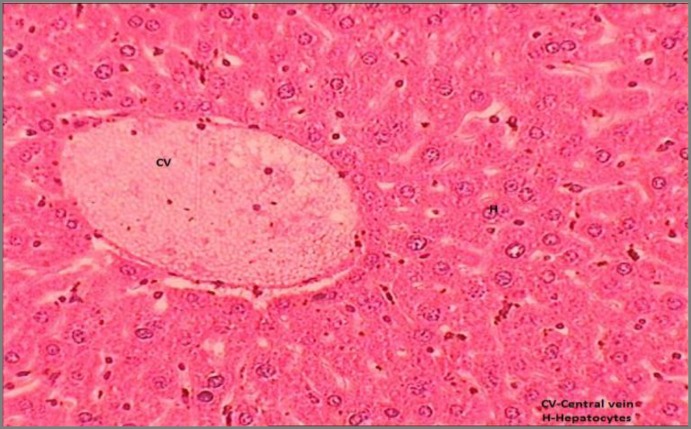
Transverse section of liver showing normal mice liver with intact central vein and hepatocytes

**Figure 5:**
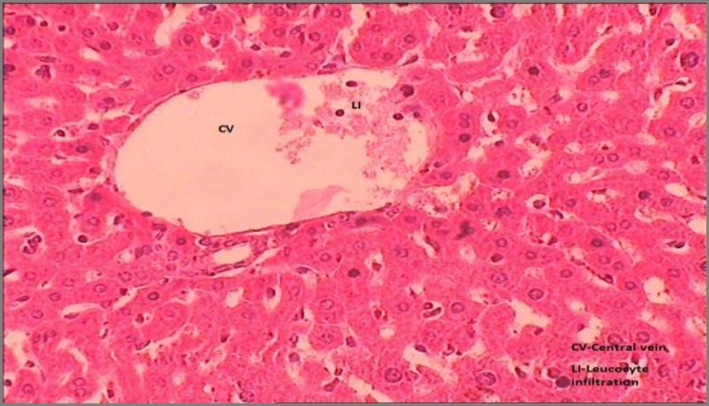
Transverse section of the liver of mice treated with NDMA showing leucocytic infiltration. CV=Central vein, LI= Leucocyte infiltration

**Figure 6:**
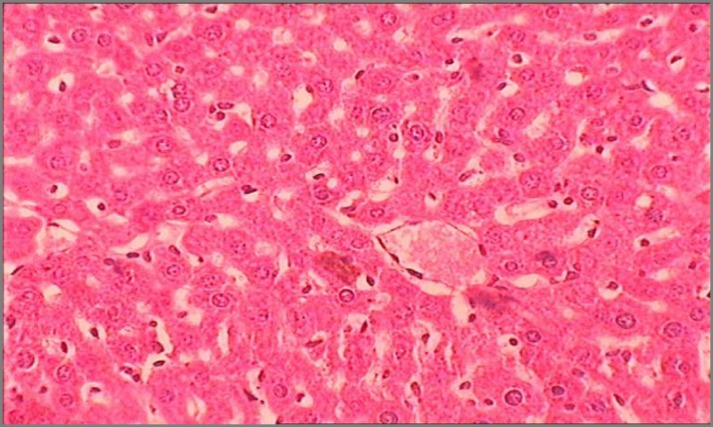
The liver showing centrilobular congestion and marked dilatation of central vein and sinusoids with massive necrosis

**Figure 7:**
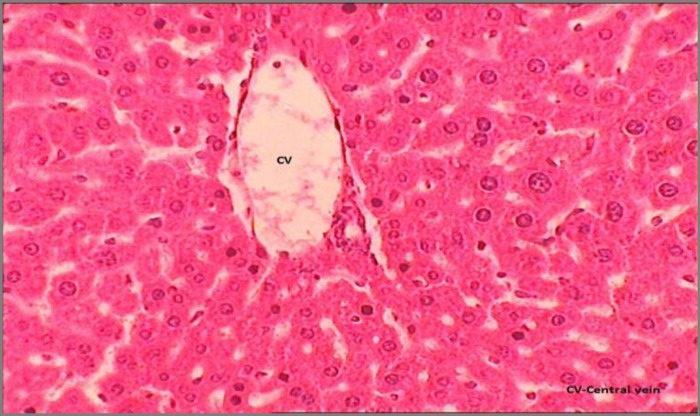
The hepatic tissue showing the regeneration whereas the central vein is still dilated

Results of histopathological studies provided supportive evidence for biochemical analysis. The histopathology reports of various groups are shown in Figures (4-7). The histo-architecture of control group (Group 1) animals was showing normal hepatocyte cells and depicted normal lobular architecture with central vein and radiating hepatic cords (Figure 4). While the animal treated with NDMA (Group 2) exhibited perivenular necrosis and micro vesicular fatty change in peripheral hepatocytes (Figure 5). There was severe centrilobular congestion and marked dilatation of central vein and sinusoids with massive necrosis and initiation of fibrosis (Figure 6). After treatment with the plant extract (400 mg/kg b.w.) the change in the liver tissue was observed including the regeneration of the normal hepatocytes (Figure 7).

The histological examination of the liver sections reveals that the normal liver architecture was disturbed by hepatocarcinogen intoxication. In the liver sections of the mice intoxicated with NDMA and simultaneously treated with the extract, the normal cellular architecture was retained as compared to NDMA treated, which confirms the protective effect of the extract. In accordance with these results, it may be hypothesized that alkaloids, saponins and flavonoids, glycosides which are present in the extract, could be considered responsible for the hepatoprotective activity.

The decrease in the necrosis area demonstrated by the extract as well as decrease in the infiltration of the inflammatory cells in the liver lobules is indicative of therapeutic efficacy of *O. turpethum*.

## Discussion

It is well known that liver toxicity is one of the most important diseases in the world. Therefore, the efforts in the hepatoprotection and treatment of liver disorders are very much required. NDMA is highly toxic to experimental animals; large single and multiple doses produce severe hepatic injuries, tumors or leukemias regardless of route of administration. NDMA reacts with rapidly proliferating cells in the terminal end buds forming DNA abducts which transform normal terminal end buds to malignant pathways. The protective effect of *O. turpethum *against DMBA-induced oxidative stress with reference to breast cancer in experimental rats has already been demonstrated (24). They suggested that the antioxidant activity of *O. turpethum* played a protective role against DMBA induced breast cancer. NDMA induced experimental hepatotoxicity might therefore be used as an ideal model to study the hepatoprotective potential of medicinal plants and their active constituents. The results reported in NDMA-induced toxicity may assist the clinician in the diagnosis, prognosis and treatment monitoring of the affected patients.

Increased oxidative stress and lipid peroxidation have been reported in NDMA induced liver injury in mice. This suggests that NDMA-induced liver damage generates free radicals, which react with polyunsaturated fatty acids of hepatic microsomal system and cause rearrangement of the double bonds to generate diene conjugated lipids (25). The improper balance between ROS metabolites and antioxidant defence results in “oxidative stress”. Participation of iron in Fenton reaction, production of more reactive hydroxyl radicals from superoxide radicals and H_2_O_2_ results in increased lipid peroxidation (26). The mechanism of free radical-induced impairment of immune system is not yet properly delineated. 

Elevated levels of enzymes are indicative of cellular leakage and loss of functional integrity of cell membrane in the liver. AST predominantly found in mitochondria of hepatocytes whereas ALT is more specific to liver, and thus is a better parameter for detecting liver injury. In the present study, NDMA was found to cause significant elevations in the levels of AST, ALT, ALP. The rise in their activities is proposed to be in good correlation with the number of damaged cells (27). Reduced glutathione, a free radical scavenger, plays a key role in the activation of T cells and macrophages. The present investigation has revealed that chronic treatment with NDMA depleted the glutathione (28).

Lipid peroxidation is a common event in any toxic phenomenon. It occurs to a limited extent under normal physiological conditions, but external factors can augment this process so that it escapes cell control which leads to damage of macromolecules such as lipids in the cell membrane and eventually causes membrane damage and death of cell. The significant depletion in the levels of TBARS in the liver tissue of the plant extract administered animal group might be due to reduced lipid peroxidation and elevation of tissue antioxidant defence enzymes activity levels, indicating that the plant extract could reduce the generation of free radicals and increase free radicals scavenging mechanism (29). The reduction in protein level attributed to the damage produced and localised in the endoplasmic reticulum which results in the loss of P_450 _leading to its functional failure with a decrease in protein synthesis and accumulation of triglycerides leading to fatty liver (30). The protein levels were increased suggesting the stabilization of endoplasmic reticulum leading to protein synthesis in animals treated with the plant extract. It can be stated that *O. turpethum* prevented an increase in cholesterol level by inactivation of thiol group enzymes such as HMG-CoA reductase and CoASH, the rate-limiting enzyme for cholesterol biosynthesis, and the multi-enzyme complex for fatty acid biosynthesis.

The reduction in liver SOD might be due to a continuous higher production of superoxide radical by the mitochondria of the damaged liver cells. Catalase converts harmful hydrogen peroxide into water and oxygen and protects the tissues from highly reactive hydroxyl radicals (31). The reduction in the activity of this enzyme may result in a number of deleterious effects due to accumulation of highly toxic metabolites and hydrogen peroxide on NDMA administration, which can induce oxidative stress in the cells (32). The increase in the levels of antioxidant profiles i.e. SOD and Catalase by *O.turpethum* extract may be attributed to its biological significance in eliminating reactive free radicals that may affect the normal functioning of cells. Liver section of *O. turpethum *treated animal group clearly showed normal hepatic cells and central vein thereby confirming the antihepatotoxic efficacy of the plant.

## Conclusion

Our data lends support to the treatment of hepatic damage by potent carcinogen NDMA in the liver of mice. The results obtained from this study prompt further study of the mechanism of antihepatotoxic activity of the root extract of *Oturpethum *at the molecular level. The plant treatment restored normal lobular architecture of the liver through extensive regeneration of hepatocytes. These attributes may provide the rationale for the use of *O.turpethum* in hepatotoxicity management in traditional medicine.
